# Traumatic Rift: How Conspiracy Beliefs Undermine Cohesion After Societal Trauma?

**DOI:** 10.5964/ejop.v15i1.1699

**Published:** 2019-02-28

**Authors:** Michal Bilewicz, Marta Witkowska, Myrto Pantazi, Theofilos Gkinopoulos, Olivier Klein

**Affiliations:** a Faculty of Psychology, University of Warsaw, Warsaw, Poland; b Oxford Internet Institute, University of Oxford, Oxford, United Kingdom; c School of Psychology, University of Surrey, Guildford, United Kingdom; d Faculty of Psychology and Education, University Libre de Bruxelles, Bruxelles, Belgium; University of Belgrade, Belgrade, Serbia

**Keywords:** victimhood, collective trauma, conspiracy beliefs, social distance, Smoleńsk

## Abstract

Collective traumas may often lead to deep societal divides and internal conflicts. In this article, we propose that conspiracy theories emerging in response to victimizing events may play a key role in the breakdown of social cohesion. We performed a nationally representative survey in Poland (N = 965) two years after the Smoleńsk airplane crash in which the Polish president was killed, together with 95 political officials and high-ranking military officers. The survey found that people endorsing conspiratorial accounts of the Smoleńsk catastrophe preferred to distance themselves from conspiracy non-believers, while skeptics preferred greater distance to conspiracy believers. We also examined the role of people’s belief in the uniqueness of in-group historical suffering as an important antecedent of both conspiracy thinking and hostility towards outgroups (conspiracy believers and non-believers).

"Don't wipe your treacherous mugs with the name of my late brother. You destroyed him, you murdered him!" – said the leader of the ruling Polish party, Jarosław Kaczyński, in a heated parliamentary debate in July 2017, while addressing the opposition party leaders. With these words he referred to the alleged responsibility of the former liberal government for the 2010 airplane crash in which the then President of Poland, Lech Kaczyński, was killed. Today, the two sides of the political debate in Poland still cannot agree upon the nature of the traumatic event that happened more than eight years ago.

Psychological research on large-scale societal traumas suggests that societies exposed to traumatic events may become more cohesive. Societal traumas can increase altruism and prosocial behavior (Staub & Vollhardt, 2008; Vollhardt, 2009), enhance ethnocentric responses (Fritsche, Jonas, & Kessler, 2011), and boost nationalism and patriotism (Skitka, 2005). Linguistic studies of on-line journals after the September 11^th^ attacks point to increased social references immediately after the collective trauma occurred (Cohn, Mehl, & Pennebaker, 2004). On the other hand, it is obvious that such traumatic events often lead to deep societal divides and intragroup conflicts – particularly when they occur in the political domain. The crash of the airplane carrying the Rwandan president Juvenal Habyarimana and the Burundian president Cyprien Ntaryamira led to the Rwandan Genocide and a violent war in the Democratic Republic Congo. The September 11^th^ attacks in the USA enhanced decades-long partisan conflict between Democrats and Republicans. The massacre in the Beslan school in 2004 escalated Ingush-Ossetian hostilities and led to human rights abuses in Russia. This societal process, known as the “cycle of violence” (Littman & Paluck, 2015), begs for a deeper explanation, particularly as far as its psychological foundations are concerned.

In this article, we analyze the key role of conspiracy theories in the breakdown of social cohesiveness after collective traumatic events. We suggest that large-scale societal traumas (such as terrorist attacks, wars, or the assassination of political leaders) can lead to disintegration and societal divisions due to the heightened need for a satisfactory explanation. When people confront large and significant events, they seek explanations that would be proportional to them in terms of significance and scale (Fiedler, Freytag, & Unkelbach, 2011). Therefore, large and significant events like accidents or disasters of national significance are more likely to elicit causal explanations that are less based on chance and rather entail the intentional activities of some individual or group. In other words, collective traumas are especially likely to elicit conspiratorial explanations, because the assumption that they are the mere result of random events is not psychologically satisfying (Klein, Van der Linden, Pantazi, & Kissine, 2015). Conspiracy theories seem to address this need, especially for those people who construe their social identity through membership in groups that are eternally victimized and the target of conspiracies and aggressive acts (Noor, Shnabel, Halabi, & Nadler, 2012; Schori-Eyal, Klar, Roccas, & McNeill, 2017). What is more, in becoming advocates of such theories, people may start considering non-believers to be potential enemies posing a threat to their epistemic certainty. Conspiracy non-believers, on the other hand, may tend to distance themselves from conspiracy believers, whom they take to be “carriers” of contagious, irrational interpretations of the traumatic event. Ultimately, a society suffering from a collective trauma may become highly polarized over these different interpretations of the traumatic event, thereby undermining social cohesiveness and leading to internal conflicts and clashes.

## Conspiracy Theories and Their Consequences

The vast majority of psychological research on conspiracy theories has focused on the antecedents of people’s tendency to believe in conspiracies (Douglas & Sutton, 2011; Swami, 2012; van Prooijen & Jostmann, 2013) and the individual-level correlates of this tendency (Abalakina-Paap, Stephan, Craig, & Gregory, 1999; Bruder, Haffke, Neave, Nouripanah, & Imhoff, 2013; Goertzel, 1994). People who believe in conspiracies are less trustful, more insecure, and more anomic (Goertzel, 1994), alienated, hostile (Abalakina-Paap et al., 1999), uncertain (van Prooijen & Jostmann, 2013) and relatively deprived (Bilewicz & Krzeminski, 2010), while they lack a sense of (socio-political) control (Bruder et al., 2013; Whitson & Galinsky, 2008). However, little is known about the consequences of conspiracy beliefs – both at the individual and the societal level. The few studies testing the consequences of conspiracy theories have shown that conspiracy theories breed feelings of powerlessness, uncertainty, and disillusionment, that in turn may lead to decreased political engagement, lower pro-environmental behavior (Jolley & Douglas, 2014a), and lower vaccination intentions (Jolley & Douglas, 2014b).

Several studies suggest that the endorsement of conspiracy beliefs by community members might be associated with lower levels of social cohesiveness within the community. When societies are confronted with a large-scale traumatic event, conspiracy-driven explanations of the event will be shared by part of the population, while at the same time other people will not endorse such beliefs. One could expect that due to mistrust, alienation, and deprivation, conspiracy believers would distance themselves from non-believers, thereby creating a rift in the societies affected by the traumatic event.

## Exclusive Victimhood as an Interpretative Framework

Clearly, people differ in how prone they are to be drawn to conspiracy theories when faced with collective traumatic events. In addition to the many individual psychological factors responsible for the endorsement of conspiracy beliefs mentioned above, collective traumatic experiences encourage the use of larger collective narratives about the in-group’s historical position in order to explain current events. Such narratives, known as “group charters” (Liu & Hilton, 2005), define the historical role of a given national group as that of historical victims, conquerors, heroes or villains. The role of historical victim could especially motivate the tendency to explain contemporary traumatic events by means of conspiracy theories.

Psychological research has identified many facets of perceived in-group victimhood, such as competitive victimhood (Noor, Brown, & Prentice, 2008), perpetual in-group victimhood (Schori-Eyal, Klar, & Ben-Ami, 2017), and exclusive victimhood consciousness (Vollhardt, 2015; Vollhardt & Bilali, 2015) that may play a destructive role in intergroup relations. All these facets of victimhood share a crucial feature, namely, the perception of the in-group as being unique in its victimhood. Past literature has shown that the perception of the in-group through such lenses of uniqueness affects intergroup processes, such as reconciliation or forgiveness (Noor et al., 2008). However, it is also likely that in-group victimhood affects the interpretation of political events and general levels of mistrust. One such effect can be, for example, that people who endorse narratives based on the uniqueness of past in-group suffering will be more inclined to believe in conspiracy theories regarding contemporary events.

## Current Study: The Smoleńsk Plane Crash as a Societal Trauma

Societal trauma is defined in social and political psychology “as an experience that invalidates one’s normal assumptions of order, predictability, safety, and identity, a very severe environmental challenge calling for the utmost energization of coping resources” (Suedfeld, 1997, p. 850). Here, we would like to focus on a recent case of such a socially shared traumatic experience for Polish society – the Smoleńsk airplane crash in 2010.

On April 10, 2010, a Polish Air Force flight crashed near the city of Smoleńsk, Russia. Ninety-six officials who were traveling to commemorate the 70^th^ anniversary of the Katyń massacre died in the accident, including the Polish president, Lech Kaczyński and his wife, the former President of Poland Ryszard Kaczorowski, several Polish government officials, top military officers, and key politicians from both the political right and left (Harding, 2010). This sudden loss of the political and military elite created a sense of threat and instability among Poles. In the days following the crash, many spontaneous and organized acts of mourning took place throughout the whole country, including thousands of candles being lit in front of the Presidential Palace in Warsaw, soon to become the center of these commemorative practices. Since the crash in 2010, every month a group of mourners assembles in front of the Presidential Palace to commemorate the late president, and to call for an investigation into the causes of the crash.

The desire to explain the catastrophe has created a rift in Polish society. In a short time, the catastrophe became a breeding ground for conspiracy explanations (Borger & Pidd, 2011). The belief in Russian sabotage as the cause of the crash and the potential involvement of the Polish liberal government therein (the so-called “Smolensk Conspiracy”), has been endorsed by 25% of Poles and has been particularly popular among right-wing voters (see Grzesiak-Feldman & Haska, 2012). Two years after the catastrophe, in 2013, we decided to perform a large-scale survey in order to assess how explanations of the airplane crash in Smoleńsk might have affected the cohesiveness of Polish society. Specifically, we wanted to assess whether the conspiratorial vs. non-conspiratorial interpretations of this event may enhance social distancing between conspiracy believers on the one hand and conspiracy non-believers on the other.

We also sought to investigate the psychological antecedent of the relationship between conspiracy beliefs and intergroup hostility. Insofar as endorsement of conspiracy beliefs is associated with beliefs about the uniqueness of in-group suffering (Witkowska, Bilewicz, Pantazi, Gkinopoulos, & Klein, 2018), we predicted that these beliefs would foster acceptance of conspiracy explanations of current events, which, in turn, would lead to the derogation of in-group members who do not share conspiracy convictions. We tested these assumptions on a nation-wide representative sample of Polish citizens measuring the belief in the *Smolensk Conspiracy*.

## Method

### Participants and Procedure

Data were collected as part of a 2013 nation-wide representative Polish survey (*N* = 965). 48.4% of the respondents were men and 51.6% were women (with an age range from 18 to 88, *M*_age_ = 46.05, *SD* = 17.52). In order to obtain an appropriate sample, participants were drawn from the population of Polish adults registered in PESEL – the Common Electronic Population Evidence System. They participated in face-to-face computer-assisted interviews, conducted by a leading Polish public opinion research institution (the Public Opinion Research Center, CBOS). Not all participants responded to all questions and the pattern of missing values did not seem to be related with any of the measured variables. In correlation and t-test analyses, the pairwise missing data deletion has been used. The SEM model we ran normally assumes the Full Information Maximum Likelihood technique of treating missing variables. However, due to missing data in the predictor variables it could not be implemented, therefore 65 cases have been deleted.

### Design and Measures

Unless otherwise indicated, participants reported their responses on 5-point scales (from 1 = “*I strongly disagree*” to 5 = “*I strongly agree*”).

***Belief in unique in-group victimhood*** was assessed with the question: “Do you agree that no other nation suffered as much as Poles did.”

In order to measure group-related conspiracy beliefs, we constructed a scale of ***Belief in the Smolensk conspiracy*** (α = .74). The scale consisted of four items that were based on the current public discourse about the Smolensk catastrophe, e.g. “Polish and Russian authorities jointly conceal the truth about the catastrophe,” and “The Smolensk catastrophe was just an accident, the result of a combination of many independent factors” (reversed).

***Social Distance towards conspiracy non-believers*** (α = .86) concerned attitudes towards people who did not endorse the conspiratorial explanation of the Smolensk catastrophe. This construct was captured by three items on a social distance scale (Bogardus, 1925). Participants had to indicate on a scale (from 1 = “*I would highly accept*” to 4 = “*I would highly oppose*”) how they would react if a person who thinks that Lech Kaczyński [the Polish President] died due to an accident moved in next door, if a close relative of theirs married such a person, or if such a person was hired by the same company as the one in which the participant works. The scores were reversed, so that higher scores on the scale meant greater social distance towards the target group.

***Social Distance towards conspiracy believers*** (α = .89) concerned attitudes towards people who believe that a conspiracy was the cause of the Smolensk crash. It was captured by adapting the items of the measurement of distance towards conspiracy non-believers.

## Results

Table 1 presents the descriptive statistics of all the variables and their correlations. The average belief in the Smolensk conspiracy was lower than the scale’s midpoint, *t*(894)= -11.93, *p* < .001. The same applied to the belief about uniqueness of in-group suffering, *t*(913) = -5.35, *p* < .001. With respect to social distance, participants’ average answers expressed general closeness to, rather than distance from, both target groups.

Social distance towards conspiracy non-believers was positively related to the belief in the uniqueness of in-group victimhood and to the endorsement of the Smolensk Conspiracy, while only the belief in the Smolensk Conspiracy correlated with social distance towards conspiracy believers, as Table 1 shows.

**Table 1:** Correlations and Descriptive Statistics

Variable	1	2	3	4
1. Unique VC (1-5)	2.76 (1.36)			
2. Smolensk Consp (1-5)	.09*	2.56 (1.11)		
3. Dist Non-Believers (1-4)	.15***	.12**	1.48 (0.59)	
4. Dist Believers (1-4)	.06^+^	-.15***	.64***	1.57 (0.69)

*Note. N* = 965. Means and standard deviations (parenthesized) reported in the diagonal. The other values represent Pearson correlation coefficients. The parentheses next to the scales’ name represent the scale’s range. Unique VC = Belief in unique in-group victimhood; Smolensk Consp = Belief in Smolensk conspiracy; Dist Non-Believers = Social Distance towards conspiracy non-believers; Dist Believers = Social distance towards conspiracy believers.

^†^*p* < .06. *p < .05. **p < .01. ***p < .001.

In order to deepen our understanding of differences between individuals endorsing and not endorsing the Smoleńsk conspiracy theory, we dichotomized the Smolensk conspiracy variable into people below and above the median. This technique allowed us to directly compare groups of conspiracy believers and non-believers in terms of their desired social distance and convictions about in-group victimhood. A series of *t*-test revealed significant differences between conspiracy believers and conspiracy non-believers (see Table 2). Individuals endorsing Smoleńsk conspiracy, proved more prone to claim uniqueness of in-group victimhood and to distance themselves from conspiracy non-believers, compared to those not endorsing this belief, but express less distance towards conspiracy believers. The longest social distance was observed among conspiracy non-believers toward conspiracy believers, followed by the distance of conspiracy believers toward conspiracy non-believers.

**Table 2:** Mean Differences Between Individuals Endorsing and Not-Endorsing Belief in Smoleńsk Conspiracy

Variable	Conspiracy non-believers		Conspiracy believers		*t*	*df*	*p*
	*M*	*SD*	*M*	*SD*			
Unique VC	2.63	1.38	2.89	1.33	-2.86	858	.004
Dist Non-Believers	1.39	0.48	1.58	0.66	-4.94	822.06	< .001
Dist Believers	1.64	0.72	1.52	0.64	2.64	851.62	.008

*Note.* Unique VC = Belief in unique in-group victimhood; Dist Non-Believers = Social Distance towards conspiracy non-believers; Dist Believers = Social distance towards conspiracy believers.

**Figure 1:**
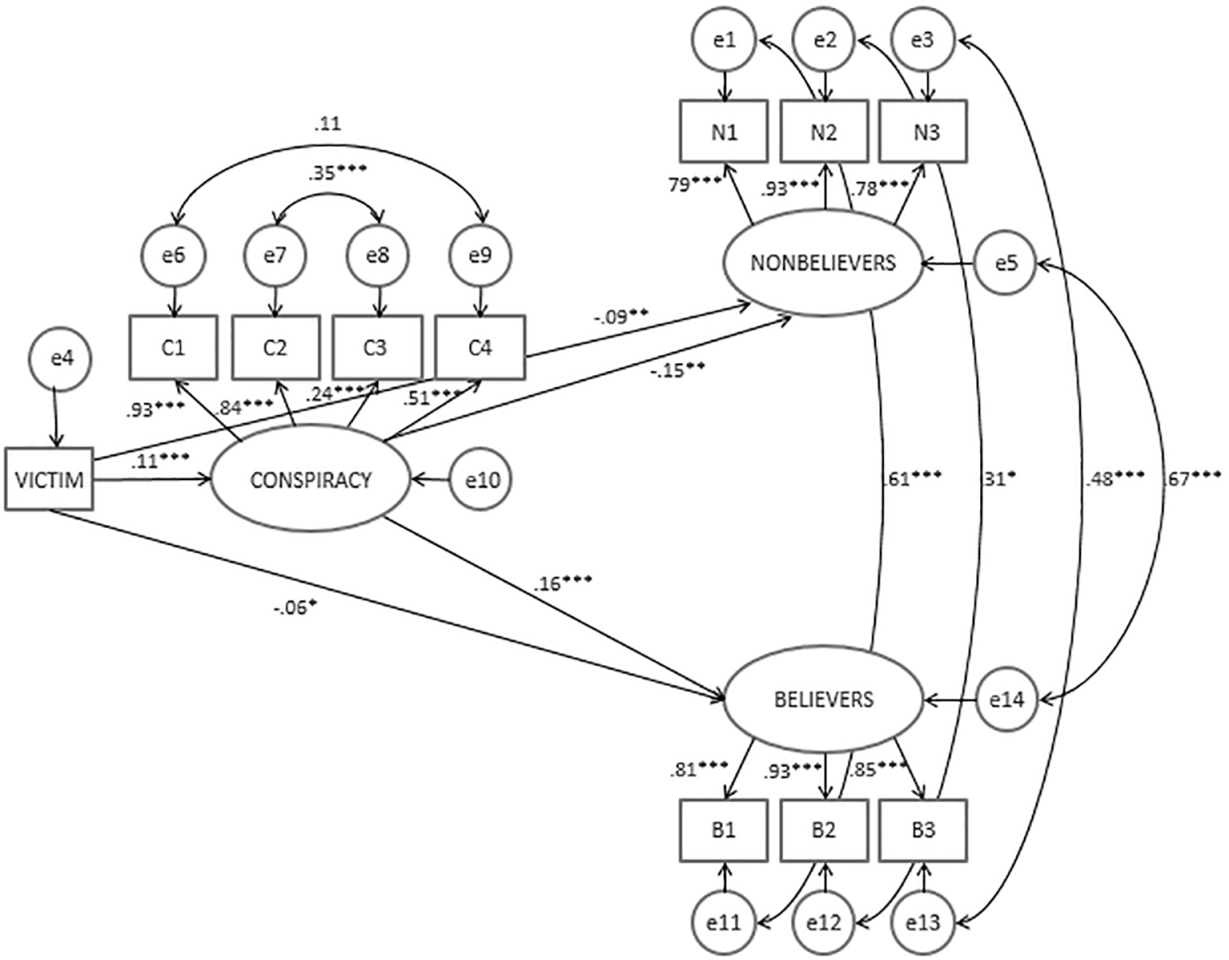
Structural equation model: Indirect effects of unique in-group suffering beliefs on distance towards Smoleńsk conspiracy believers and non-believers through the endorsement of conspiracy beliefs of the air crash. Standardized regression coefficients. *p < .05. **p < .01. ***p < .001.

To integrate the analyses for the two indices of social distance we specified a structural equation model using MPlus 6 software (see: Figure 1). In this model we used the four items tapping into Smoleńsk conspiracy scale as indicators of a latent conspiracy beliefs variable. Social distance towards conspiracy believers and social distance towards conspiracy non-believers were introduced as latent variables with three indicators each. Belief in unique in-group victimhood was treated as a manifest variable.

We estimated a model in which beliefs in unique in-group victimhood was regressed on latent conspiracy beliefs and both latent social distance variables. The latent social distance constructs were also directly predicted by latent conspiracy beliefs. The model included covariance between the two latent social distance variables. The model also reflected patterns of question format as we allowed for covariance between series of indicators variables (i.e. in the Smoleńsk conspiracy we allowed for a correlation between two positively formulated items and between two negatively formulated items; in the social distance scales, items capturing the same level of social distance like neighborhood or common working place were correlated). The model exhibited acceptable fit, χ^2^(34) = 121.52, *p* < .001, CFI = .98, RMSEA = .05, SRMS = .042. Endorsement of unique in-group victimhood was associated with the latent conspiracy belief construct which in turn predicted the two social distance latent variables. The direct link between unique victimhood belief and both latent constructs of social distance was also significant. We then tested for indirect effects of belief in unique victimhood on latent social distance variables. We found a relatively weak but significant indirect effect of IE = 0.003 for distance towards conspiracy nonbelievers, *z* = 2.47, *p* = .01, 95% CIs [0.00, 0.005]. The indirect effect on distance towards conspiracy believers was not significant, estimate = -0.003, *z* = -1.81, *p* = .07, 95% BCIs [-0.01, 0.001] (see Figure 1).

The test of the causal structure between in-group victimhood (unique in-group suffering beliefs), conspiracy beliefs about Smoleńsk air crash and social distance towards people endorsing and not endorsing conspiracy beliefs, suggests that the general sense of victimhood affects distancing from the conspiracy non-believers through higher endorsement of conspiracy theories among those who consider their in-group suffering to be unique. At the same time the conspiracy theories of societal trauma lead to greater societal distancing between believers and non-believers.

## Discussion

The present survey study performed in the aftermath of the Smoleńsk catastrophe suggests that conspiratorial explanations of this collectively traumatic incident undermined social cohesion: People endorsing conspiratorial accounts of the Smoleńsk catastrophe expressed the desire to distance themselves from conspiracy non-believers, whereas people opposing conspiracy explanations preferred greater distance to conspiracy believers. The analyses of social distance between conspiracy believers and conspiracy non-believers suggest that those who do not endorse conspiratorial explanations are more willing to distance themselves from their opponents, compared to the distance that conspiracy believers desire. In other words, the results of belief vs non-belief in conspiracy theories may have disproportional effects on social distancing from out-group (skeptics vs. conspiracists) members, potentially because skeptics consider conspiracy thinking contagious and more dangerous, than conspiracists consider skepticism. This interpretation is supported by a recent study showing that conspiracy beliefs may be socially stigmatized (Lantian et al., 2018). Thus, there seem to be alternative reasons for non-believers to distance from the proponents of conspiracy theories, one of them could be the fear of contamination (Haidt, Rozin, McCauley, & Imada, 1997) supported by the stigmatizing nature of conspiracy belief.

As expected, higher belief in unique in-group suffering was found to account for conspiracy thinking and subsequent hostility towards the outgroup, as defined by belief in conspiracies or not. This result is consistent with previous research showing that beliefs concerning the uniqueness of in-group trauma are related to greater suspiciousness in intergroup relations (Bar-Tal, Chernyak-Hai, Schori, & Gundar, 2009). Therefore, historical charters that position the group in the position of the victim create an interpretative framework for subsequent socially shared traumatic events. People who believe in the unique victimization of their nation are more likely to endorse conspiracy beliefs and this is likely to enhance their social distance to non-believers. To sum up, three broader statements underpin our findings: Firstly, views about national history affect contemporary responses to traumatic events. Second, people focused on historical victimhood interpret current traumas in a conspirational way and distance themselves from skeptics. Third, people skeptical about conspiracy explanations of trauma distance themselves from conspiracy believers even more strongly than those who do endorse such explanations distance themselves from skeptics.

The phenomenon of social distance (Bogardus, 1925) is often treated as an indication of prejudice and discriminatory intention. However, it might also indicate a more general tendency to isolate oneself from other people, regardless of their views and group memberships. The positive relationship between social distance toward conspiracy believers and non-believers at least partly suggests such a possibility. In order to overcome this issue, we decided to treat the errors of social distance scales as correlated, which allowed us to directly assess the effects of conspiracy endorsement on distance toward believers vs. non-believers (as opposed to a general tendency to isolate oneself).

There are some inherent limitations to the present investigation. Fist, the effect sizes we obtained for all the observed effects suggest that there is still a vast amount of investigation needed in order to fully understand the interplay of conspiracy theories and social cohesion. The correlational effects are relatively low, as is the variation of the key dependent variable. This might be due to the fact that traditional social distance measures are imperfect measures of distancing intentions in politicized conflicts (as opposed to ethnic or religious ones). In such conflicts, it might be unwillingness to accept someone as a boss, priest or a representative that is more representative of distance, rather than unwillingness to accept such person as a neighbor.

Second, due to the correlational nature of the data collected, these results are obviously restricted and should be treated with caution as for the causal structure of the phenomenon. Given the inability of mediation analysis to identify unique mediators (Fiedler, Schott, & Meiser, 2011), our analysis cannot be seen as statistically proving the accuracy of our mediation hypothesis. It is worth highlighting, however that the proposed casual path, whereby beliefs about the uniqueness of in-group suffering facilitate the endorsement of conspiracy explanations of current events, is based on extended theoretical elaboration and also supported by other experimental research (Witkowska et al., 2018). Further research is needed to address this controversy by means of experimental tests of mediation analysis, or a longitudinal design (Bullock, Green, & Ha, 2010). In any event, the present study has the advantage of offering an insight into a unique period of Polish history when the country seemed to be split over the explanation of a socially shared traumatic event.

Overall, the findings reported in this article provide a novel explanation for the negative behavioral consequences of victimhood beliefs for intragroup attitudes that occur in the situation of a large scale societal trauma. Specifically, they showed that historical group charters may induce conspiracy believers to distance themselves from the non-believers when they interpret traumatic events. This might be due to the specificity of societal traumas in political domain that seems substantially different from individual traumas or natural disasters that often bring social cohesion rather than divide. Future empirical studies, potentially based on computer simulations using agent-based models (Eberlen, Scholz, & Gagliolo, 2017), could further examine the dynamics of societal divisions after political traumatic events.
